# Current News and Opinion

**Published:** 1882-07

**Authors:** 


### CURRENT NEWS AND OPINION.

DR. HENRY J. BIGELOW has resigned the Chair of Surgery in the Medical Department of Harvard University.

''A SYSTEM of Gynecology by American Authors'' is to issue from the press of Henry C. Lea's Son & Co. early next spring.

ANOTHER death from chloroform in a dentist's chair. This time in Birmingham, Ala. Isn't it about time some one were punished for such wicked carelessness?

AN ASSOCIATION for the advancement of medicine by original research has been formed in England. It strikes us that there is room for such a society on this side of the water.

MEDICINE IN JAPAN.—Japan possesses 159 hospitals. Vaccination is performed gratuitously, and is compulsory. Persons without certificates are prohibited from practicing medicine or surgery.

BEEF TEA AND URINE.—An analysis of beef tea and urine shows these to be chemically identical, and Dr. Neale, of London, recommends the latter as a vehicle for the more disagreeable medicines.

THE AMERICAN Surgical Society met in Philadelphia May 31 and June 1, and was better attended than the previous meetings. Several excellent papers were read. Prof. S. D. Gross was re-elected President for the current year.

BEARDED INFANTS.—The *Tiempo* of Valencia notes the birth of an infant with a well developed beard and a full set of teeth. Our dental colleagues will note this fact and give Valencia a wide berth; it may however be a good place for barbers.

Female Oculists.—The *Uniao Medica* tells us, among a quantity of equally interesting and reliable statistics, that the United States possess four eminent female oculists. The journal does not say what their personal appearance is, nor whether they employ the ophthalmoscope in the direct method for determining errors of refraction.

It is proposed to raise 100,000 dollars in order to endow a chair for Prof. Joseph Leidy, who has been Professor of Anatomy in the University of Pennsylvania for thirty years. The amount is to be raised by subscription. We know no one in this country more deserving of such a substantial acknowledgment for services rendered to science.

Nearly a Century and a half.—The *Uniao Medica* of Rio de Janeiro extracts the following from the *Gazeta Paranaense*: "In Piedade, District of Votuverava, Francisco Bueno da Rocha lives. He is 133 years of age and in perfect health. A grandchild of one of his grandchildren is a marriageable woman." And he was not G. Washington's body servant !

Assistant Wanted.—Professor Dr. Flechsig advertises that applications may be addressed to him for the position of first assistant to the clinic for the Insane of the University of Leipzig. The salary is 1800 Mark (1 M. = 25 c.) and board and lodging. It is about time that some Americans go to Europe to teach them what they don't know about neuropathology.—"What *who* don't know, etc., and *who* to teach *whom*?" Hazard of the St. Louis *Clinical Record* will ask ambiguously.

(Diese Anzeige kostet 4 M. die Zeile. Man bittet um gefællige Remesse.)

Errare humanum est.—Dr. L. Lœwenfeld of Munich, in an article on "Causes of transitory psychical disturbances," in the *Neurologisches Centralblatt*, tells of a lady patient who, when suffering from one of these conditions, frequently called him (the Dr.) "thou," and mistook him for her husband.—Get the journal, naughty young Dr.'s, read the article carefully, and when you feel like degrading the noble profession into which you sneaked, by telling of your amorous exploits, remember that perhaps the poor victim of your misdirected attentions was suffering from a transitory psychical disturbance, and that your charms had no part in the conquest (?).

"El Medico y Cirujano Centro Americano."—This journal has not come to us for some time, but its editor and proprietor, Dr. Ferd. C. Valentine, did. He told us that the journal is "not dead but sleeping,"

to reawaken shortly with its office in the United States. Brother Valentine has done good work in the Tropics, and will continue his successful efforts to draw practice and trade from the Tropics to the United States, which, before his advent in Central and South America, went to Europe. Bien venido, Don Fernando ! His office is at 30 West Eleventh St., where he tells physicians and dentists all about good locations in the Tropics, and shows them his collections of Indian curiosities.

